# Supplementary material for: High Predictive Value of the Soluble ZEBRA Antigen (Epstein-Barr Virus Trans-Activator Zta) in Transplant Patients with PTLD
Source: Pathogens. 2022 Aug 17;11(8):928. doi: 10.3390/pathogens11080928 (PMC9413454; doi:10.3390/pathogens11080928)

Supplementary data

Centre	Transplantation	Number of samples (of cases)
A	Pulmonary	42 (14)
B	Renal	23 (12)
C	HSCs	132 (61)
D	Pulmonary, renal, HSCs	24 (13)
**TOTAL:**		221 (104)
E	HSCs	139
	Renal	70
	Cardiac	49
	Pulmonary	33
	**TOTAL:**	291

Table S1: Case and control patients enrolled in the study: Retrospective case collection yielded 221 plasma samples from 104 patients, from the hospitals of Nantes, Strasbourg, and Utrecht (Netherlands) and from the Cryostem biobank (Marseille). However, 69 patients (142 samples) were excluded due the lack of clinical/anatomopathological data. Four centers provided a total of 221 plasma samples from 104 patients. A: Nantes University Hospital, B: Strasbourg University Hospital, C: Cryostem, D: UMC Utrecht, E: Grenoble University Hospital, HSCs: Hematopoietic stem cells.

**Cells (time of harvesting)**	**Absorbance (OD 450 nm)**	**ZEBRA amount in cell supernatant (ng/mL)**
Non treated Akata Cells	0,01	0
AKATA cells treated by anti-Ig (12h)	0,71	120
AKATA cells treated by anti-Ig (48h)	1,50	220

Table S2: Detection of ZEBRA in the supernatant of induced Akata cells. Akata cells were maintained in RPMI 1640 medium supplemented with 10% fetal bovine serum, penicillin, 50 U/ml, streptomycin, 50 U/ml, and amphotericin B, 1g/ml. Induction of ZEBRA was performed by cross-linking the B-cell antigen receptor: anti-IgG (Dako) was added at 7.5 µg/mL to induce the lytic cycle (according to [50]). Supernatant was harvested by 12 h and 48h after anti-IgG treatment and soluble ZEBRA was quantified by ELISA test (in µg /mL). Annexin V assay was negative at 12h post induction and positive at 48h post induction.

Figure S1: Calibration curve to obtain the direct correlation between OD450 and the concentration of sZEBRA, in the ELISA.

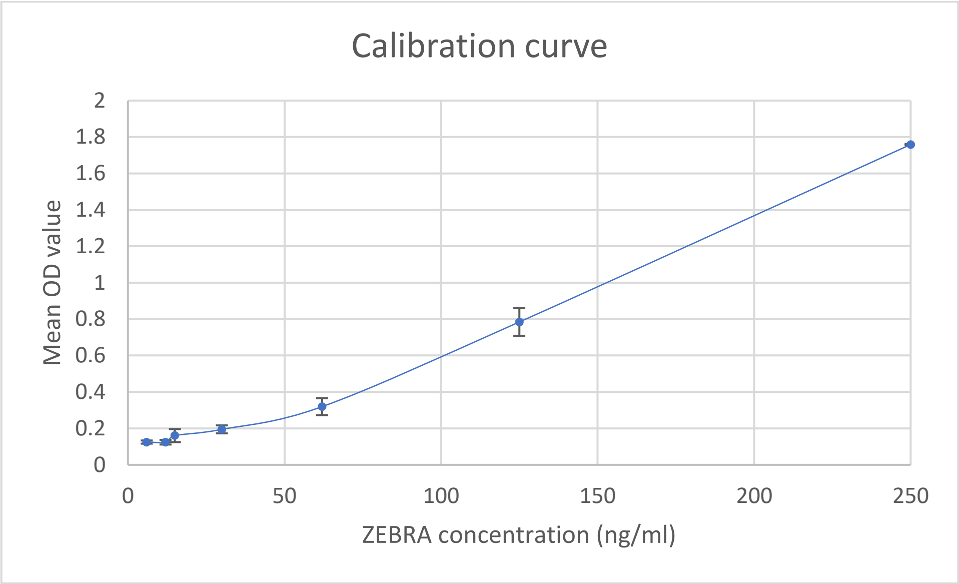

Supplement: Supplementary file 1 [file pathogens-11-00928-s001.zip › pathogens-1658846-supplementary-8.17.pdf]
